# A method to determine spatial access to specialized palliative care services using GIS

**DOI:** 10.1186/1472-6963-8-140

**Published:** 2008-06-30

**Authors:** Jonathan Cinnamon, Nadine Schuurman, Valorie A Crooks

**Affiliations:** 1 Department of Geography, Simon Fraser University, 8888 University Drive, Burnaby, British Columbia, V5A 1S6, Canada

## Abstract

**Background:**

Providing palliative care is a growing priority for health service administrators worldwide as the populations of many nations continue to age rapidly. In many countries, palliative care services are presently inadequate and this problem will be exacerbated in the coming years. The provision of palliative care, moreover, has been piecemeal in many jurisdictions and there is little distinction made at present between levels of service provision. There is a pressing need to determine which populations do not enjoy access to specialized palliative care services in particular.

**Methods:**

Catchments around existing specialized palliative care services in the Canadian province of British Columbia were calculated based on real road travel time. Census block face population counts were linked to postal codes associated with road segments in order to determine the percentage of the total population more than one hour road travel time from specialized palliative care.

**Results:**

Whilst 81% of the province's population resides within one hour from at least one specialized palliative care service, spatial access varies greatly by regional health authority. Based on the definition of specialized palliative care adopted for the study, the Northern Health Authority has, for instance, just two such service locations, and well over half of its population do not have reasonable spatial access to such care.

**Conclusion:**

Strategic location analysis methods must be developed and used to accurately locate future palliative services in order to provide spatial access to the greatest number of people, and to ensure that limited health resources are allocated wisely. Improved spatial access has the potential to reduce travel-times for patients, for palliative care workers making home visits, and for travelling practitioners. These methods are particularly useful for health service planners – and provide a means to rationalize their decision-making. Moreover, they are extendable to a number of health service allocation problems.

## Background

Providing palliative care has become a priority for health system administrators worldwide. In many countries, existing palliative care service delivery is inadequate and challenged by population aging, resulting in strained services that are increasingly unable to keep up with demand [1-4]. There is also growing realization that the development of palliative care services in many jurisdictions has been piecemeal, unplanned, and largely unregulated which has ultimately exacerbated disparities in access to receiving care by geographic location and socio-economic status [5-7]. At the same time, it is recognized that initiating palliative care for a patient with a life-limiting illness can reduce the burden placed on health care systems through freeing up space in acute care settings and halting curative treatments when the prognosis becomes terminal [8]. Furthermore, it is recognized that patients should have the right to receive care that is appropriate to their needs [9-11] and not be limited to simply what is available. As a result of these factors, health systems in Canada and worldwide are starting to recognize the need for improved palliative care delivery [6,10,12,13].

The equitable provision of health care services (i.e., care that is offered in a fair and just manner based on need) in an accessible fashion is a primary concern for health planners; however, 'access' can be defined in many different ways. Access to health services can, for example, refer to: availability in terms of socio-economic status or geographic location, the availability of information, wait-list times, and quality of services offered [4,14,15]. Health systems must therefore take into account various aspects of accessibility when planning service delivery. Recognition that health care services are not equally or equitably accessible by all has started to drive policy making in palliative care [16]. In British Columbia (BC), the most western province in Canada, there is increasing recognition of the need for better spatial access to palliative services for all residents. A recent framework for end-of-life care created by the BC Ministry of Health outlines a commitment to "establishing high quality end-of-life care and support as an integral part of our provincial health system" [17]. The need for palliative services to be accessible to all residents of the province, and as close as possible to their home location is highlighted. Services located near to palliative care recipients' home communities will not only improve spatial accessibility for the patient, but will also reduce travel-time for workers making home visits and practitioners that deliver care in multiple communities. The present study examines the spatial accessibility of palliative care services for the residents of BC by employing a novel spatial analysis approach using Geographic Information Systems (GIS). The study focuses on specialized palliative care (SPC) services, but the spatial methodology introduced is relevant to a number of health service allocation problems in multiple jurisdictions.

### Spatial Accessibility of Palliative Care

The influence of location and distance to health services on health outcomes are well documented [18-21]. Home location has been shown to determine access to and utilization of health services, with utilization being inversely related to spatial access [14,20,22]. Furthermore, it has been argued that a person's home location can also be a determinant of his/her overall level of health [23]. Thus, people who reside closer to sites of care delivery are more likely to utilize these services and obtain better health outcomes as a result. Spatial proximity to health care services particularly affects the elderly and younger populations, individuals with physical and mental impairments, and those residing in rural and remote areas through reduced mobility associated with these populations [22,24-26].

Canadian health organizations have expressed the need to research the geographic and demographic factors that influence palliative care delivery and uptake [27,28]. GIS-based analyses can provide robust decision support for health service studies by uncovering the geographic and demographic determinants of service utilization. An increasingly popular use of GIS in this realm is to determine the spatial accessibility of services based on distance or travel-time from residence to site of care. Straight-line and road-network based measures of distance from residence to site of care have been used in the past to measure accessibility. However, these methods are inappropriate for modelling access in large regions like BC because of the geographic diversity characterized by mountain ranges, valleys, meandering coastlines, and a mix of urban centres and rural hinterland. Various methods of measuring travel-time have been used in modelling spatial accessibility to health care services [see [19,29-33]]. Haynes *et al*. [34] validated the use of travel-time to measure spatial accessibility by comparing a GIS-based travel-time model with actual driving time to service locations. Results were highly correlated for modelled and actual travel-time to health care service locations.

In Canada, provincial health systems are obligated to ensure access to health services for all citizens, based primarily on the principles of 'universality' and 'accessibility' which are enshrined in the Canada Health Act [15]. GIS methods allow for evidence-based planning that can promote universally accessible health care services. In this study we determine the current spatial accessibility of palliative care services in BC using a proven vector GIS catchment method [32]. This method is appropriate for studying access to health services in geographically diverse regions such as BC because it is based on road network travel-time from home to location of care. This study presents a description of those areas of BC that are within one hour travel-time to SPC services and the proportion of the population that existing services are reaching. Identification of communities that are greater than one hour from services is provided to shed light on possible locations where new palliative services could be implemented to service rural and remote areas in particular.

### Specialized Palliative Care

There is no single, universally accepted understanding of what palliative care services or practice entail [12,35]. This has contributed to wide disparities in the resources, capacity, and infrastructure devoted to palliative care delivery [1], and poses questions and challenges for palliative care research. As such, diversity is a hallmark of palliative care, evidenced by the range of treatments, sites of delivery, and populations eligible for care. Traditionally palliative care was directed at relieving pain in patients with cancer in the final stage of illness, though increasingly it is becoming available to those who require care for longer periods at end-of-life as well as those with non-cancerous conditions [2,36-38]. The type of palliative care delivered varies from pain management and comfort care to therapies and spiritual counselling [39]. Counselling, bereavement services, and respite care for patients' families have also entered the palliative care service basket. Palliative care can be delivered by specialized practitioners, general practitioners, allied health professionals, and informal caregivers such as volunteers and family members [25]. The care itself can be delivered in hospice residences, acute care settings, care facilities, and patients' homes [17].

The vagaries associated with palliative care can act as roadblocks to the effective promotion and delivery of quality end-of-life care [40]. What is needed, then, is an inclusive definition. Definitions posited by the World Health Organization (WHO) have been refined several times, mirroring the increased attention on end-of-life issues. The current WHO definition [41] is as follows:

Palliative care is an approach that improves the quality of life of patients and their families facing the problems associated with life-threatening illness, through the prevention and relief of suffering by means of early identification and impeccable assessment and treatment of pain and other problems, physical, psychosocial and spiritual.

Based on suggestions from the European School of Oncology, Ahmedzai *et al*. [42] refined the WHO definition in a way that could help to improve both service delivery and research into palliative care issues. Palliative care can be usefully sub-divided into basic and specialized categories (p. 2194).

*Basic palliative care *is the standard of palliative care which should be provided by all healthcare professionals, in primary or secondary care, within their normal duties to patients with life-limiting disease.

*Specialised palliative care *is a higher standard of palliative care provided at the expert level, by a trained multi-professional team, who must continually update their skills and knowledge, in order to manage persisting and more complex problems and to provide specialised educational and practical resources to other non-specialised members of the primary or secondary care teams. If a patient has difficult symptoms which cannot be controlled by his/her current healthcare team, he/she has a right to be referred, and the current healthcare provider has an obligation to refer, to the local palliative care team. [42]

This distinction between basic and specialized care is useful to categorize the different types of care and locations of palliative care service delivery. Care that is delivered by general practitioners, some allied health professionals, and informal caregivers in home and acute care settings can be considered basic palliative care. Multi-focal care which is delivered by a multi-disciplinary team with some palliative care specialization in a variety of settings including the home constitutes SPC. Patients receiving SPC often have access to a wider variety of specialist treatments and support, from comfort care to intense pain management, while their families can access a host of services that can include respite care, counselling and bereavement services. These services are provided by specially trained experts, which helps to ensure the best possible quality of death and dying for patient and support for the family during the palliative stage.

Albeit not definitive, studies have found that SPC is more effective at improving patients' quality of life in their final days than basic palliative care [43-45]. Also, a study by Morita *et al*. [46] found that bereaved family members of cancer patients who had received SPC were happier with the services provided compared with those who had not. Furthermore, general practitioners have acknowledged the benefits of SPC and favour referring a dying patient to a specialist at the end-of-life if this service is available [47,48]. Ensuring access to SPC for palliative care recipients should be prioritized because it stresses education for all stakeholders and the right to expert care for all as a human right [1]. Reasons for establishing and/or enhancing SPC are well-grounded and the benefits have been demonstrated; however, it is impossible to have palliative experts in all communities, especially in rural and remote areas. According to the SPC model, specialists should deliver palliative care whenever possible, but in communities where SPC is not feasible, local general practitioners and informal and voluntary-sector caregivers should be trained in specialized methods.

The purpose of this study is to model spatial accessibility to palliative care in British Columbia using the SPC definition. Accessibility is determined through calculating the proportion of the total population that is within a reasonable travel-time to a SPC location. Also, geographically distant communities without SPC services are highlighted as potential candidate locations to provide SPC to surrounding rural and remote areas. The SPC definition was chosen because it provides a standard of comparison for future spatial analyses of palliative care services in other countries and regions. Furthermore, it provides a framework for our future research goals of determining appropriate locations for siting regional hubs of palliative care to serve the rural and remote regions highlighted by this study. These hubs will boast multi-professional teams to deliver SPC and will have the ability to educate local practitioners and caregivers in remote areas to provide good-quality basic palliative care where SPC is not feasible.

## Methods

This study calculates the service areas or catchments of existing SPC locations in BC and the populations within those service areas. At the same time, the study identifies and calculates populations outside one hour to SPC. In addition, proportions of the population within and without the service catchments are calculated. The locations of current SPC services in the province were collected by directly contacting each of the five regional health authorities. The locations that adhered to this definition included hospice residences and designated palliative care units within hospitals with a minimum of five beds. Five beds was considered the minimum for a hospital palliative unit because it was expected that this would also mean the unit would have access to a multi-disciplinary team of palliative experts. Some smaller hospitals in BC set aside one or two beds for palliative patients on an as-needed basis, though care for these patients is typically provided by non-specialized volunteers and practitioners, and as such, do not meet the criteria for SPC. Thus, the number of beds was used as a proxy indicator for the presence of an on-site multi-disciplinary team of palliative care specialists and not an indicator of palliative care supply.

Locations of SPC services in each of the five BC health authorities were geocoded and mapped using ArcGIS 9.2 [49]. To create the service areas for each location, network-based travel-time catchments were created using the Network Analyst extension in ArcGIS, based on a proven travel-time catchment method [32]. Such an approach is part of an established literature on the use of travel-time to measure spatial accessibility [see [29-31,50]]. The Road Atlas of BC dataset from GIS Innovations was used to provide travel-time measurement along road networks. This extensive road dataset includes travel impact variables such as speed limits, road surfaces, and stop signs for each road segment which allows for an accurate prediction of travel-time between any two points in the province that are connected by the road network. Catchments were created based on a travel-time of one hour to service location. One hour travel-time was chosen as it reflects a reasonable daily commute to and from sites of care for palliative care workers and informal caregivers, because permanent relocation to urban areas is inconsistent with the wishes of those with a life-limiting illness or their families[51].

To determine the population within each catchment, a spatial query method was employed using census block-level population data [52], the finest-scale at which population data is available in Canada. The total population of all individual Census Block geographic units within a maximum distance of 2500 metres from the catchments were summed to determine the total population that is within one hour drive to service locations. Schuurman *et al*. [32] describe the catchment creation and population linking procedures in greater detail in a study of access to hospitals in BC. Communities outside of the one-hour service areas were highlighted using a spatial query that selected all census-defined urban areas (communities with at least 1000 residents, with a population density of at least 400 people per km^2^) that had centroids within 2500 metres of the catchments.

## Results

**Figure 1:**
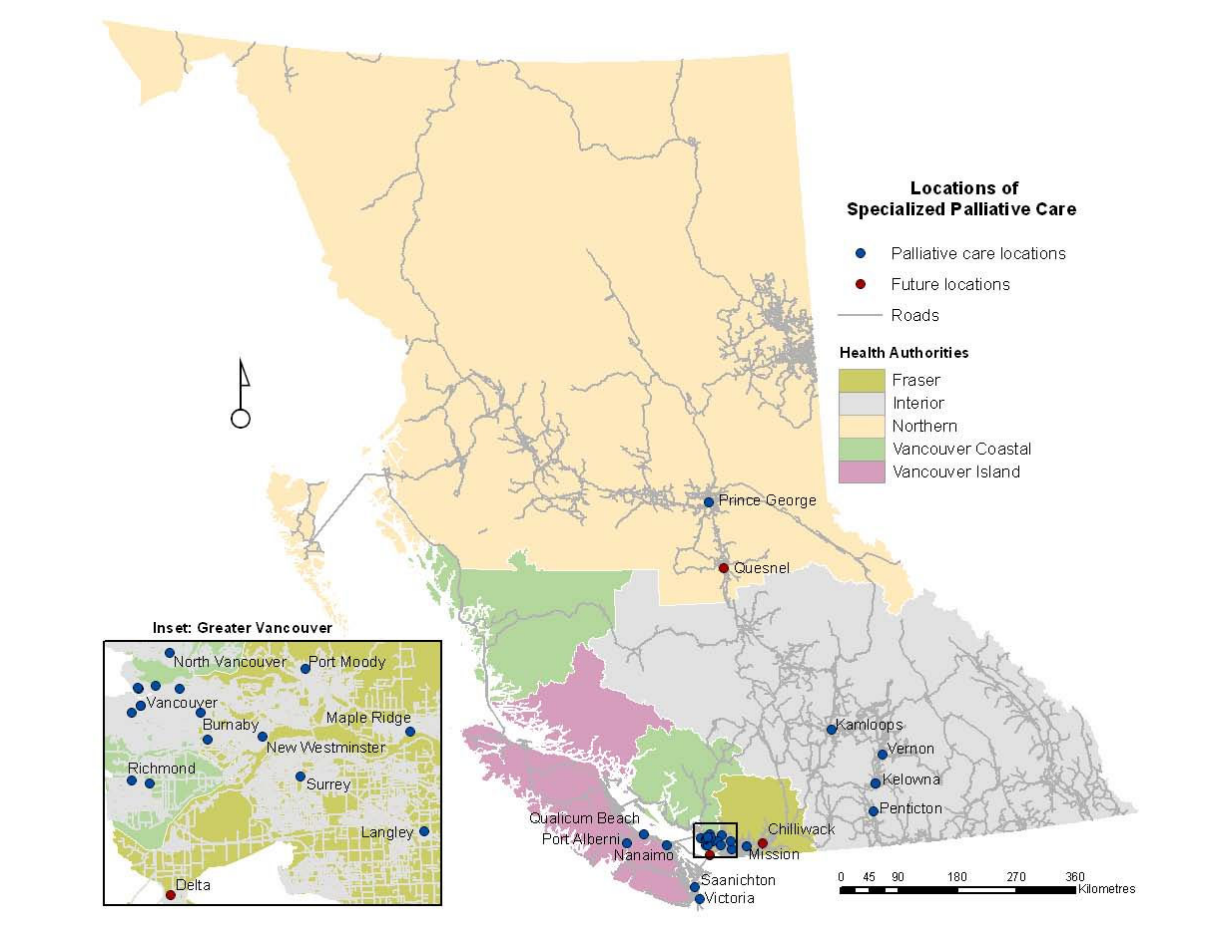
**Map of British Columbia showing locations where SPC is delivered**. Currently 29 locations in BC offer SPC. Three new locations are scheduled to open in 2008–09.

**Figure 2:**
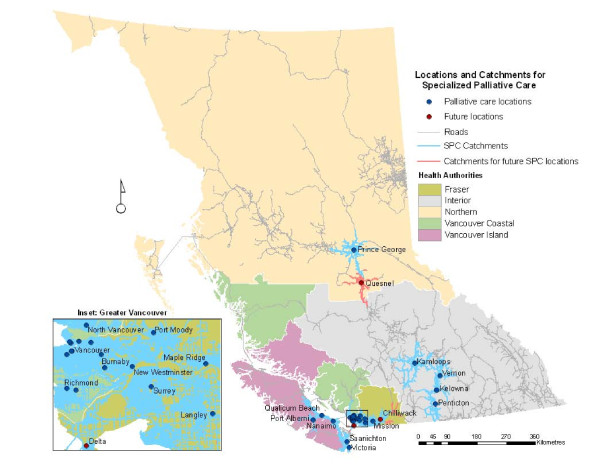
**One hour travel-time catchments for existing and future SPC locations in British Columbia**. Catchments show the service areas for existing and future services. The farthest reaches of the catchments indicate the location that is exactly one hour drive to the nearest SPC service location.

According to our working definition at the time of the analysis there were 29 locations where SPC was delivered in BC and three new locations slated to open in the near future. The sites of delivery included hospice residences and palliative care units in hospitals, each with varying capacities. Figure 1 shows the communities where SPC services are delivered in BC. One-hour travel time catchments for SPC services are shown in Figure 2. Catchments were created for all 29 existing and three future service locations.

Table 1 summarizes the number of service locations and the total population of each health authority. Also shown is the total population and percentage of the population that has access to SPC for each health authority. The results include the three new service locations that are scheduled to open in 2008 or 2009. The Northern Health Authority (NHA) had the lowest proportion of its population within one-hour to SPC at 36%, based on just one existing location in Prince George and one future location in Quesnel. Just 60% of the Interior Health Authority (IHA) had access to SPC from five service locations. More than 95% of the population of the Fraser Health Authority (FHA) lived within one-hour of its eight current and two future locations. Almost 90% of the Vancouver Coastal Health Authority's (VCHA) population was within an hour drive to SPC based on ten locations. Five locations in the Vancouver Island Health Authority (VIHA) served almost 84% of its total population.

**Table 1:** Palliative Care Locations and Population within Catchment Areas for each Health Authority

**Health Authority**	**Locations**	**Total Pop.**	**Pop. In Catchment**	**% in Catchment**
FHA	8 (+2)	1,387,010	1,326,967	95.67
IHA	5	683,863	411,150	60.12
NHA	1 (+1)	297,415	107,150	36.03
VCHA	10	1,016,380	909,121	89.45
VIHA	5	693,779	582,100	83.90
**Total**	**29 (+3)**	**4,078,447**	**3,336,500**	**81.81**

Total population figures from Statistics Canada (2002).

**Figure 3:**
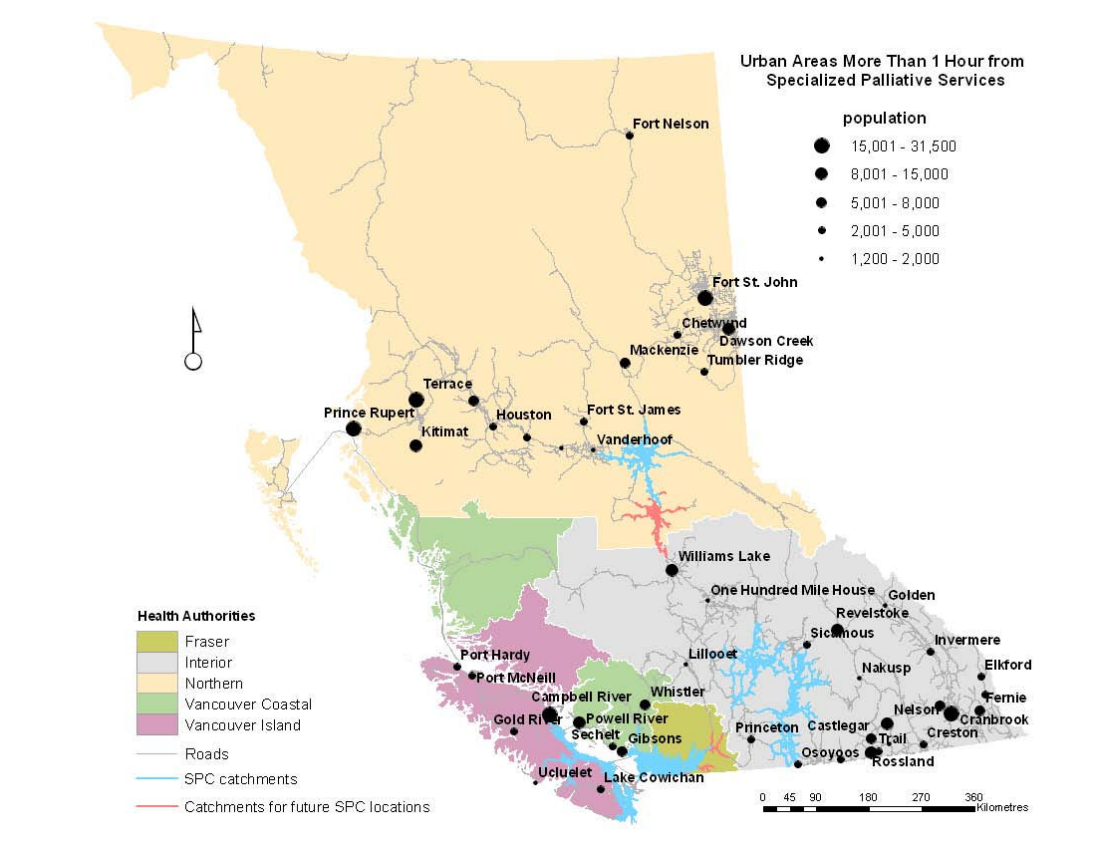
**Urban Areas outside of the one-hour SPC catchments**. Fifty communities of varying size are more than one hour from SPC services. The largest communities considered to be out of service range are Campbell River, Cranbrook, Prince Rupert, Terrace, and Fort St. John.

All of the Urban Areas greater than one-hour from SPC services are highlighted in Figure 3. IHA had the greatest number of urban areas (25) outside the one-hour catchments. The VCHA had four, VIHA had six, and NHA had 15 Urban Areas that were greater than one hour from SPC. All of the urban areas in the FHA were within one hour travel-time to SPC.

Several larger regional centres are without access to SPC. Table 2 lists the largest communities in each health authority outside of the one-hour catchments. In the NHA, Terrace, Prince Rupert, and Fort St. John each have a population greater than 15,000 and are well outside of the SPC service areas. In the IHA, residents of Revelstoke, Williams Lake, and several larger communities in the Kootenay region are without reasonable spatial access to SPC services, including Nelson, Trail, Castlegar, and Cranbrook. Whistler and Powell River are the largest communities in the VCHA region that do not have access to SPC. Campbell River, with a population of over 31,000 in the VIHA, sits just outside the existing SPC catchments. Further, several other smaller urban areas in more remote parts of Vancouver Island are at a significant spatial disadvantage for accessing such services.

**Table 2:** Largest Communities Greater Than One Hour to SPC by Health Authority

**Health Authority**	**Urban Areas**	**Population**
FHA	N/A	0

IHA	Cranbrook	18,131
	Williams Lake	12,621
	Nelson	9,585
	Trail	9,484
	Revelstoke	8,042
	Castlegar	7,610
NHA	Terrace	17,596
	Prince Rupert	16,633
	Fort St. John	15,021
	Dawson Creek	11,125
	Kitimat	10,551
VCHA	Powell River	13,131
	Whistler	7,112
VIHA	Campbell River	31,038

Source: Statistics Canada, 2002.

## Discussion

We have undertaken a spatial analysis of spatial access to SPC services in BC. Figure 1 reveals that the majority of SPC locations are clustered in the extreme south-west of the province in and around the Vancouver area. This is no surprise given that a majority of the province's population resides in this area. Further, the FHA is recognized as having significantly invested in developing palliative care service infrastructure within its catchment area. The remaining locations are dispersed in the southern half of BC, mainly in larger regional communities.

At first glance, it appears that the spatial accessibility of SPC in BC is reasonably good, with over 81% of the province's population residing within one hour from at least one service location. Whilst this is true, spatial access varies greatly by health authority as illustrated in Table 1 and Figure 2. The NHA has the lowest population base, but it is spread over the largest geographic area. With just two SPC locations, well over half of its population do not have reasonable spatial access to such care. The situation in the IHA is similar with just over half of its population enjoying access to SPC. Most of the residents in the VCHA and FHA are within an hour drive to one or more SPC sites. Relatively good spatial access to SPC in these two heavily populated health authorities skews the overall rate to make it appear that access is reasonably good for the province as a whole.

By determining areas that are outside of the catchments, we can identify regions for which health system administrators should consider siting further SPC services in the province. Figure 3 highlights the urban areas in BC that are without spatial access to SPC services, specifically those located greater than one-hour of driving time away from sites. Of the 93 Statistics Canada defined urban areas that exist in BC, 50 are greater than one hour from the nearest service location. Evidenced by the above analysis, NHA and IHA communities are particularly burdened by unequal spatial access to SPC. The communities listed in Table 2 are potential candidates for situating future SPC services. The largest communities are most suitable because a large and stable core population is needed in order to ensure enough demand and also justify expenditures. Specifically, larger communities such as Prince Rupert in the NHA, and Cranbrook or Campbell River in the IHA are the most likely candidates based solely on their core population sizes. Surrounding populations must also be taken into consideration when analyzing site suitability in order to achieve economies of scale. Several clusters of larger communities are evident in Figure 3. Clustering is apparent in the north-west NHA, including Prince Rupert, Terrace, and Kitimat, and in the north-east with Dawson Creek, Fort St. John, and Chetwynd. The Cranbrook-Fernie-Kimberley and Nelson-Castlegar-Trail areas in the Kootenay region of the IHA are in close proximity to each other. Installation of SPC in one community in each cluster would likely ensure spatial access to SPC for the residents of all of the nearby communities and rural areas. The next stage of this research is to assess the suitability of these sites for situating SPC services. The age structure and other relevant socio-demographic characteristics of each community will be taken into consideration to create a robust index of site suitability based on potential need.

### Limitations

Use of the SPC definition care may discount the efforts of informal caregivers and non-specialized types of care which also likely benefit the patient at the end-of-life. However, despite focussing on specialized care, this study does acknowledge the need for basic palliative care in some communities where SPC is not available or feasible. Another potential limitation was the fact that the supply side was considered to be equal among centres, and demand for SPC was not addressed. Several GIS modelling types are available which take supply and demand into consideration; however, these methods are not appropriate for palliative care. Supply is usually measured as the number of care beds or number of physicians. As palliative care consists of a diverse set of therapies and interventions delivered in a multitude of settings, it is likely impossible to determine the exact supply of services for any given area. In terms of demand, we assumed the whole population of the study area to be potential users of SPC. This is a reasonable assumption given that this type of care is now targeting all age groups; the only restriction is that a patient must have a life-limiting condition.

Possible limitations regarding the use of one hour as a reasonable travelling distance to services are apparent. This travel-time was chosen because of its importance within emergency services as the 'golden hour', the period of time in which patients in need of emergency care should arrive at hospital to minimize the risk of serious consequences [53,54]. One hour has been used in other GIS-based spatial analyses of travel-time to health services in large regions [29,32]. Furthermore, a sensitivity analysis helped to confirm the appropriate choice of catchment size for this study. Catchments based on a 90 minute travel-time captured less than 2.9% (minimum 0% in FHA, maximum 8% in VIHA) more of the province's population overall compared with the 60 minute catchments. The original 60 minute catchments captured over 5.5% (minimum 0.6% in FHA, maximum 18.6% in VIHA) more than 30 minute catchments. Thus, the sensitivity analysis was useful in confirming our choice of one hour as the threshold for this study, as the increase in population was greater from 30 to 60 as opposed to the smaller gain from 60 to 90 minute catchments. True, larger catchments would likely suggest greater spatial accessibility, though the longer commute is an increased burden for palliative patients who wish to spend their remaining days at home, and the smaller increase in population gain renders larger catchments less appropriate as a measure of accessibility in this study. The other limitation with creating catchments based on a strict travel-time threshold is that spatial access drops to zero just outside the catchment. This is the case in Campbell River which is located just outside a catchment. In reality, this community may be serviced by the SPC locations in Port Alberni, Qualicum Beach or Nanaimo. Additionally, solely considering spatial access oversimplifies a diverse concept. Despite the potential limitations, this study provides a useful analysis of the distribution of SPC in BC, the population of BC that can reasonably spatially access SPC, and has highlighted several communities where SPC implementation may benefit rural and remote regions.

### Implications

It is likely that numerous individuals at end-of-life living in communities significantly outside of the one-hour catchments either die without receiving SPC, are forced to commute an unreasonable distance to get to non-local/regional service sites, or relocate to larger urban centres that have existing SPC. Relocation, however, is often against the wishes of patients and their families and runs counter to the trend of ageing-in-place in rural and remote BC communities [55]. Furthermore, it is acknowledged that dying at home or in one's home community is preferred by a majority of people [56-58]. The need for easily accessible SPC is further heightened by the move towards providing palliative care on day visits and short-term admissions for symptom control and not as a form of residential care [38,59-63]. Therefore, reasonable daily travel to and from a SPC site for the care recipient and his/her family, or to-and-from the patient's home or home community for a SPC professional, is crucial. If a patient is accepted into a hospice residence or other residential service site (e.g., acute care hospital), it is vital that family and friends be able travel regularly to visit and provide comfort in his/her final days. Finally, if a patient is able to remain at home during his/her final days and requires only minimal therapeutic intervention (e.g., pain management, wound care), it is imperative that service providers are available within a reasonable travel-time from the care recipient's home to deliver them.

According to our model, close to three quarters of a million people in BC (almost 20%) lack access to SPC, and in general, spatial access is non-existent for people who do not live in the Vancouver region or the larger urban centres on Vancouver Island and in the province's interior. Unique challenges exist in delivering a full complement of health and social services in rural and remote BC [55]. Policies are beginning to change as rural and remote regions adopt new models of service delivery that reflect local needs. We acknowledge that implementing SPC in every community is not feasible given resource constraints and the sparse and widely-distributed nature of the BC population. The creation of sub-regional centres is a possible solution to delivery problems in rural and remote regions, if strategically planned [64,65]. Decentralization has occurred throughout BC through devolution of control to the regional health authorities [66,67], though, we argue, this has not led to greater accessibility for many types of services including palliative care. Strategically located sub-regional care facilities could facilitate spatial access to services within a reasonable travel time, and may better positioned to assess and respond to the unique needs of local rural and remote areas than provincial or sub-provincial resource dissemination centres. In the case of palliative care in BC, sub-regional care facilities could disseminate information and resources to the local region and provide education for patients, families, and general practitioners while being accountable to the larger health authorities. These facilities could also ensure home-based and non-specialized types of palliative care services are of a consistent quality, at least at the local level. This may mean an expansion of the role of current SPC locations (hospice residences and palliative care units) to include regional regulation and educational capacities. New facilities should be developed with this holistic approach in mind that includes delivery, education, and regulation of SPC.

## Conclusion

Providing equitable spatial access to good quality palliative care is a growing priority for health systems worldwide. Distance and location in part determine utilization of services and influence health outcomes. Unique challenges for palliative care delivery exist in BC as a result of the large rural and remote areas where services are often limited, and distance to locations with specialized services are great. GIS methods can be used to model health service catchments, thereby highlighting the populations that have access to care. This study used a vector GIS-based method to identify areas of BC that are within one hour travel-time to SPC services and the proportion of the population in each provincial health authority that existing services reach. A working definition of palliative care was formulated to facilitate the modelling and analysis, and to promote further spatial analyses of palliative care. Also described were the communities that are more than one hour from services.

Using the working definition, 29 existing SPC and three future locations (hospice residences and palliative care units) were identified. Creation of catchments for all 32 locations illustrates that over three-quarters of BC's residents are within one-hour drive to SPC. However, distribution varies greatly by health authority, ranging from just 36% in the NHA to 95% in the FHA. A majority of the communities outside of the one-hour catchments are located in either the NHA or IHA. The larger (e.g., Cranbrook, Campbell River, Terrace, Fort St. John) and more clustered communities (north-west, north-east, south-east) are posited to be potential locations where new SPC services could be implemented to improve spatial access for those residing in local rural and remote areas. Current and future locations of such services should focus on delivery, education, and regulation of SPC.

Strategic location analysis methods must be developed and used to accurately locate new SPC services to in order to provide spatial access to the greatest number of people. Specifically, more research is needed into the factors and constraints that ultimately determine the suitability of locations to host SPC than what is provided in the present study. Characteristics of future locations that should be considered include: the presence of other health services and infrastructure, availability of support services, and population demographics, among other factors. Such consideration will work to ensure that limited health resources are allocated wisely.

Whilst the information gleaned from this study is important for planning of palliative care services, the methodology is also extendable to other health services. It provides a means of rationalizing service allocation based on maximizing the number of people served within a designated road travel time.

## Abbreviations

SPC: specialized palliative care; BC: British Columbia; IHA: Interior Health Authority; FHA: Fraser Health Authority; NHA: Northern Health Authority; VIHA: Vancouver Island Health Authority; VCHA: Vancouver Coastal Health Authority; WHO: World Health Organization; GIS: geographic information systems.

## Competing interests

The authors declare that they have no competing interests.

## Authors' contributions

JC conducted the spatial analysis and wrote the first draft of the manuscript. NS conceptualized and developed the methodology, and assisted in writing and editing the manuscript. VAC provided domain expertise in palliative care and health services and assisted in writing and editing the manuscript.

## Pre-publication history

The pre-publication history for this paper can be accessed here:


